# The need for rejuvenation of Indian biomedical journals

**Published:** 2010-12

**Authors:** Shampa Ghosh, Jitendra Kumar Sinha

**Affiliations:** * Department of Neurophysiology, National Institute of Mental Health & Neuro Sciences, Bangalore & India; ** Endocrinology & Metabolism Division, National Institute of Nutrition (ICMR), Hyderabad, India

Sir,

Impact factor (IF) is often used as a benchmark for the relative importance of a journal within a particular discipline. The next source is the Scientific Citation Index (SCI)^1^ which provides researchers and scientists with quick access to the bibliographic and citation information they need to find relevant, comprehensive research data. SCI helps in tracking prior research work in a particular area, monitoring current developments as well as assessing the influence of our work based on observations from the citation records. The greater the use of a particular research information or idea from a publication, the higher will be its SCI. However, a paper of low IF and SCI does not necessarily mean it is of lesser use or relevance. From the dawn of the history of science, new and bold ideas have always been discouraged by the prevailing scientific community and even in this cutting-edge technological era, it is no exception- a new hypothesis is simply rejected due to lack of sufficient literature or because it comes from a not-so-famous laboratory which does not have state-of-the-art facilities.

The problem with the non-English speaking underdeveloped and developing countries is that the expression of ideas and thoughts becomes so difficult for the original researcher that often an external help is sought after to put it on paper. This is the first step where dilution of ideas happens and intellectual quality of the research article goes down. Sometimes, we also contradict ourselves- on one hand we want our journals to attain international standards but when it comes to publishing our exemplary findings, we prefer international journals to the Indian ones. This may be partially due to the ‘snail-mail’ approach for the processing of publications by most Indian journals till recent times, making the whole procedure very slow. However, many of the Indian journals like IJMR have now started an online submission system making the entire process convenient and faster for researchers.

Recently Satyanarayana & Sharma^2^ rightly portrayed some of the vital issues of the quality of biomedical journals in India. We have sister concerns of all international societies in about every field of biomedical research, but still do not have journals of international standard. Even if a research article is assessed qualitatively, leaving aside the impact factor and SCI, it is expected that at least the research output should be accessible to the scientists worldwide. Unfortunately most Indian journals are not available on the World Wide Web. It is shocking to know that only 0.71 per cent of Indian journals are on PubMed and a bit more when searched on other worldwide databases^2^. So most of the research endeavours of our country still go unnoticed and that puts us in a much lower rung of the ladder of global scientific productivity.

The editors of journals should not only be experts in the field but also well-trained in editorial responsibilities^2^. The dependability and reproducibility of the results in different research articles should always be verified by the editors, even if the paper comes from an established laboratory, as inadvertent errors can never be ruled out. The publisher and editors of journals should prioritize to get their journal indexed so that the articles are easily available through worldwide databases. But as rightly mentioned^2^, this must never be done at the cost of the quality of publication. The practical and important issues regarding publication, ethics, plagiarism, conflict of interest, and very importantly authorship has been thoroughly discussed^2^. A junior researcher, many-a-times finds his name somewhere down the long list of authors or does not find it at all, due to lack of impartial judgment of the mentors which leads to frustration and demotivation. Sometimes, it also happens that the name of the person who corrects grammatical errors in the manuscript appears before that of the primary researcher who has toiled to make the study successful. This is very unhealthy for the nascent research environment of any country. Thus, before submitting a particular research article the mentors should take proper care to encourage young researchers and bestow appropriate value to the person who has contributed the maximum towards the scientific finding. This will increase the efficiency of scientific output of our country. Apart from research publications, Ph.D. / MD thesis are being published by many foreign Universities, making the research works of the scholars accessible to the global scientific community. This should be introduced in our country also. Within a year of awarding the degrees, encourage young researchers and bestow appropriate value to the person who has contributed the maximum towards the scientific finding. This will increase the efficiency of scientific output of our country. Apart from research publications, Ph.D. / MD thesis are being published by many foreign Universities, making the research works of the scholars accessible to the global scientific community. This should be introduced in our country also. Within a year of awarding the degrees, the thesis/dissertation content may be posted on a national database (to begin with) like MedIND (*http://medind.nic.in/*). In this way, the entire thesis content (the study design, review of literature, methodology and conclusion) except for the unpublished results is within the reach of other researchers who can gain new ideas and use this information as the foundation of a new study. Also, when such a study is open to discussion and peer-review, through positive criticism, the study design can be improved and more effective protocols can be devised so that future research in the same area is more sound and effective.

Instead of pointing fingers to others, the need of the hour is to introspect – to identify the places where we are lacking transparency, to accept our limitations and understand our strengths so that we can work upon them to improve. This surely will scale up the scientific output and increase the global visibility of research work (not only in the field of biomedical sciences but also in other areas) carried out in India.
